# Development and Application of a Microfluidics-Based Panel in the Basal/Luminal Transcriptional Characterization of Archival Bladder Cancers

**DOI:** 10.1371/journal.pone.0165856

**Published:** 2016-11-15

**Authors:** Doris Kim, YounJeong Choi, James Ireland, Oded Foreman, Rachel N. Tam, Rajesh Patel, Erica B. Schleifman, Maipelo Motlhabi, Dorothy French, Cheryl V. Wong, Eric Peters, Luciana Molinero, Rajiv Raja, Lukas C. Amler, Garret M. Hampton, Mark R. Lackner, Omar Kabbarah

**Affiliations:** 1 Oncology Biomarker Development, Genentech, South San Francisco, California, United States of America; 2 Department of Biostatistics, Genentech, South San Francisco, California, United States of America; 3 Alternate Allele Consulting, Orinda, California, United States of America; 4 Department of Pathology, Genentech, South San Francisco, California, United States of America; Centro Nacional de Investigaciones Oncologicas, SPAIN

## Abstract

In the age of personalized medicine stratifying tumors into molecularly defined subtypes associated with distinctive clinical behaviors and predictable responses to therapies holds tremendous value. Towards this end, we developed a custom microfluidics-based bladder cancer gene expression panel for characterization of archival clinical samples. *In silico* analysis indicated that the content of our panel was capable of accurately segregating bladder cancers from several public datasets into the clinically relevant basal and luminal subtypes. On a technical level, our bladder cancer panel yielded robust and reproducible results when analyzing formalin-fixed, paraffin-embedded (FFPE) tissues. We applied our panel in the analysis of a novel set of 204 FFPE samples that included non-muscle invasive bladder cancers (NMIBCs), muscle invasive disease (MIBCs), and bladder cancer metastases (METs). We found NMIBCs to be mostly luminal-like, MIBCs to include both luminal- and basal-like types, and METs to be predominantly of a basal-like transcriptional profile. Mutational analysis confirmed the expected enrichment of *FGFR3* mutations in luminal samples, and, consistently, FGFR3 IHC showed high protein expression levels of the receptor in these tumors. Our bladder cancer panel enables basal/luminal characterization of FFPE tissues and with further development could be used for stratification of bladder cancer samples in the clinic.

## Introduction

Bladder cancer is the fifth most common malignancy worldwide, with close to 400,000 newly diagnosed cases and ~150,000 associated deaths per year [1, 2]. Approximately 75% of patients present with low grade, non-muscle-invasive bladder cancers (NMIBCs) at the time of initial diagnosis [3–5]. NMIBCs recur in ~50% of instances and can progress in ~30% of cases to more serious muscle-invasive bladder cancers (MIBCs) [3, 4]. Recurrence risk factors include tumor histopathological features such as tumor stage and grade [3, 4]. The presence of high risk histological characteristics often prompts physicians to recommend more aggressive therapeutic intervention, including radical cystectomy and adjuvant therapy [3, 4]. MIBCs (T2 and T3) constitute ~15% of new cases and they are more likely than NMIBCs to develop into metastatic bladder cancers (METs) (pT4) [3–5]. Although METs represents <10% of newly diagnosed cases, they are associated with a poor 5-year survival likelihood of ~15% and represent a major unmet medical need [5–9].

Transcriptionally defined classes of bladder cancers were identified and have been shown to be associated with distinctive clinical behaviors and responses to therapies [10–15]. Accordingly, transcriptional characterization of bladder cancers has the potential to complement histopathological assessment in guiding treatment decisions. Recently, Choi *et*. *al*. described subsets of MIBCs that were transcriptionally reminiscent of breast cancers, including a luminal subtype characterized by a high frequency of activating *FGFR3* mutations and a favorable outcome, and a basal subtype that exhibited a low frequency of *FGFR3* mutations and was associated with a poor prognosis [14]. Consistent with these findings, Damrauer and colleagues reported on luminal and basal transcriptionally defined subtypes of primary MIBCs that were associated with distinct clinical outcomes [10]. The significant progress in our molecular understanding of bladder cancer offers a unique opportunity to classify tumors into the clinically actionable basal and luminal subtypes. However, RNA derived from FFPE samples is of much lower quality than that obtained from frozen samples, which are typically used to derive transcriptional classifiers. Thus, a specially designed gene expression panel that is optimized for robust transcriptional characterization of archival tissues could have high clinical utility. Furthermore, basal/luminal features have only been studies in MIBCs and NMIBCs [10, 14, 16, 17], and no studies have been conducted to date on the basal/luminal characteristics of METs.

In this study, we introduce a novel microfluidics-based panel that is custom designed to measure the expression of bladder cancer-relevant genes in FFPE tissues. *In silico*, we demonstrate that the content of our panel is capable of distinguishing between basal and luminal bladder cancers. We employ our panel in the analysis of FFPE tissues from a novel cohort of 204 tumors and show that NMIBCs from our sample set are mostly luminal-like, carry *FGFR3* mutations at high frequency, and express high protein levels of the receptor tyrosine kinase. On the other hand, MIBCs from our cohort are mixed with respect to luminal- and basal-like status, and the expression patterns seen in METs are consistent with a more basal-like transcriptional state.

## Results

### Development and in silico validation of a microfluidics-based panel for transcriptional characterization of archival bladder cancer tissues

**Fig 1:**
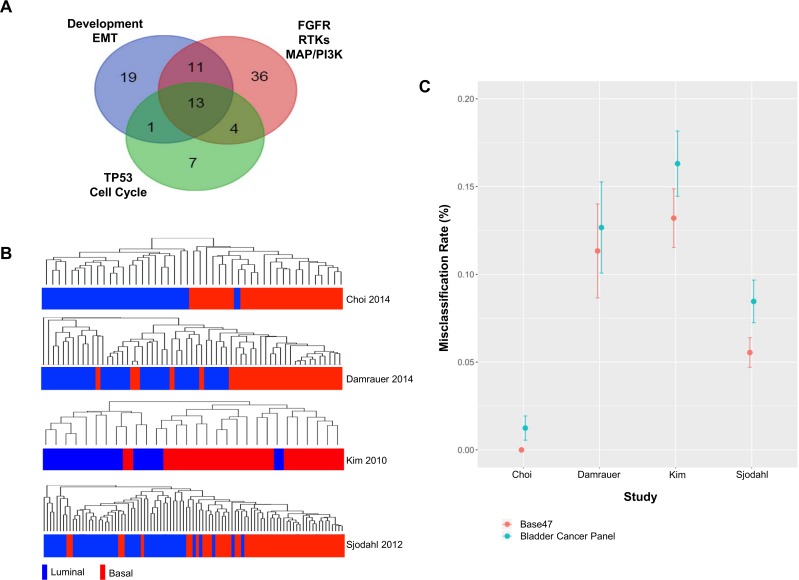
Development and *in silico* validation of a bladder cancer gene expression panel for characterization of FFPE tissues. (A) Venn diagram of genes comprising a novel bladder cancer panel illustrating the number overlapping (and unique) genes belonging to three main pathway groups based on Ingenuity® analysis: 1) FGFR, RTK, MAPK, and PI3K pathways; 2) development and EMT axes; 3) TP53, genome stability, and cell cycle regulation networks. (B) Hierarchical clustering of samples from four public datasets based on the signals from probes corresponding to genes on the bladder panel, and corresponding published basal/luminal status. (C) Basal/luminal misclassification rates represented as % of all cases in four public datasets comparing calls made by the BASE47 gene signature from literature to assignments made based on the expression of genes on the bladder panel.

Here, we introduce a microfluidics-based bladder cancer gene expression panel that is optimized for the analysis of FFPE tissues. Our panel is comprised of 90 unique genes that were selected to capture key attributes of bladder cancer biology [18–31], as well as two housekeeping genes for data normalization (Figs 1A and S1, S1 Table). The genes include components of receptor tyrosine kinase (RTK) pathways such as FGFR, ERBB, and MET, the PI3K/AKT and MAPK axes, cell cycle and genome stability genes like *TP53*, genes involved in the regulation of cell differentiation and development, and epithelial-mesenchymal transition (EMT) genes [18–31] (Figs 1A and S1, S1 Table).

Given the biological and clinical relevance of the recently described basal and luminal subtypes of bladder cancer [10, 14], we carried out an *in silico* assessment of the ability of the genes on our panel to distinguish between these two molecular subtypes in public datasets. We used samples from four public data sets that were designated as basal or luminal by Choi *et*. *al*. [14] or through BASE47 scoring by Damrauer, *et*. *al*. [10] and hierarchically clustered samples based on the expression of probes corresponding to genes on our panel (Fig 1B). The content of our panel was able to correctly segregate samples based on luminal and basal status from the Choi discovery dataset [14] in 23/24 (96%) and 23/23 (100%) of instances, respectively (Fig 1B, Table 1). Furthermore, based on our genes we could accurately separate specimens from the Damrauer discovery set [10] into luminal and basal subtypes in 33/33 (100%) and 23/28 (82%) of the cases, respectively (Fig 1B, Table 1). Finally, the content of our panel correctly distinguished between 10/12 (83%) of the luminal and 17/18 (94%) of the basal tumors from the Kim cohort [12], and 39/44 (80%) of the luminal and 42/47 (89%) of the basal samples from the Sjodhal dataset [13], as determined by Damrauer using BASE47 classification [10] (Fig 1B, Table 1). Interestingly, only 1/90 and 23/90 genes from our panel overlapped with the BASE47 Damerauer [10] and Choi [14] basal/luminal signatures, respectively (S2 Table, data not shown).

**Table 1:** Basal/luminal misclassification rates in public datasets using bladder cancer panel and BASE47 gene sets.

Public dataset	GEO accession	Sample #	# of samples with available basal/luminal calls	% of basal/luminal samples correctly classified by bladder cancer panel	BASE47 misclass rate +/- SD	Bladder panel misclass rate +/- SD
Choi et. al., 2014 (Discovery set)	GSE48075	73	23/24	100%/96%	0.000+/-0.000	0.008+/-0.006
Damrauer et. al., 2014	GSE5287	30	18/12	82%/100%	0.107+/-0.027	0.140+/-0.034
Sjodahl et. al., 2012	GSE32894	308	49/44	94%/83%	0.057+/-0.009	0.082+/-0.017
Kim et al., 2010	GSE13507	165	28/33	89%/80%	0.133+/-0.013	0.156+/-0.020
Damrauer et. al., 2014	GSE5287	30	18 / 12	82% / 100%	0.107 +/- 0.027	0.140 +/- 0.034

Next, we further evaluated the ability of the genes on our panel to distinguish between basal and luminal samples by performing classifier analysis and used cross-validation to calculate the average misclassification rates in assigning the correct basal/luminal status. More specifically, for a given literature data set samples were divided into training and test sets. A diagonal linear discriminant analysis (DLDA) classifier [32] was trained to distinguish between basal and luminal samples in the training samples based on all bladder cancer panel genes. The classifier was then used to predict whether the remaining test samples were most likely to be basal or luminal. These predictions were compared to the assigned basal/luminal status by Choi *et*. *al*. [14] and Damrauer and colleagues [10], and this information was used to calculate the basal/luminal misclassification rate. Random permutation in sample assignments to test and training sets was repeated until all samples had been included as a test sample one time. Classifier analysis was performed for each public data set separately. Using this approach, we observed similar basal/luminal misclassification error rates for our bladder cancer panel compared to assignments made using the BASE47 gene set in the Choi discovery samples [14] (Fig 1C, Table 1). We also observed comparable basal/luminal misclassification rates for the Damraurer samples [10], as well as for samples from the Sjodhal [13] and Kim [12] data sets (Fig 1C, Table 1). These results indicate that the content of our panel is capable of classifying MIBCs into basal and luminal subtypes with similar accuracy to that obtained by the BASE47 gene set.

Our initial cross-validation classifier analysis demonstrated that the full content of our panel allowed for accurate classification of samples from four different public datasets into basal and luminal subtypes (Fig 1C, Table 1). However, the panel contains genes related to diverse bladder cancer pathways not all of which may be important in distinguishing between basal and luminal cancers. We investigated whether subsets of the genes on our panel could inform basal/luminal status in the public datasets. We used a gene ranking system based on Welch’s t-test and incorporated a gene selection step as part of the cross-validation process where only the top 10, 20, 30, 40, 50, 60, 70 and 80 most differentially expressed genes between basal and luminal samples were included in the classifier. We found that as few as 20 genes could classify samples with similar misclassification rates compared the full panel (S2 Fig). This observation was consistent across all four public data sets (S2 Fig). Using the entire set of samples from each of the four public datasets, we then calculated the Welch’s t-test statistic and p-value significance levels for all genes on our panel. This allowed us to rank all the genes on our panel and select a smaller subset of genes that were significantly differentially expressed between basal and luminal bladder cancer for future studies (S2 Table).

We next explored if the genes on our panel could classify cancers into disease-relevant subtypes beyond the basal and luminal groups. The cohort described by Choi *et*. *al*. [14] contained basal and luminal MIBCs as well as tumors that exhibited a p53-like signature that were resistant to frontline chemotherapy based on transcriptional data from frozen tissues [14]. Hierarchical clustering of samples from the Choi [14] cohort demonstrated that the expression of genes on our panel was able to correctly classify 20/24 (83%) luminal, 22/23 (96%) basal, and 17/26 (65%) p53-like samples (S3 Fig).

The findings from our *in silico* validation analysis suggest that the genes on our panel are capable of classifying tumors into basal and luminal subtypes with similar accuracy to the public classifiers, which were specifically developed to differentiate between basal and luminal samples. Furthermore, our panel was capable of differentiating between p53-like and basal/luminal bladder cancer samples, albeit without the same degree of accuracy as when distinguishing between basal and luminal samples. Thus, we focused on applying our panel in basal/luminal characterization of bladder cancers.

### Technical validation of the bladder cancer panel

**Fig 2:**
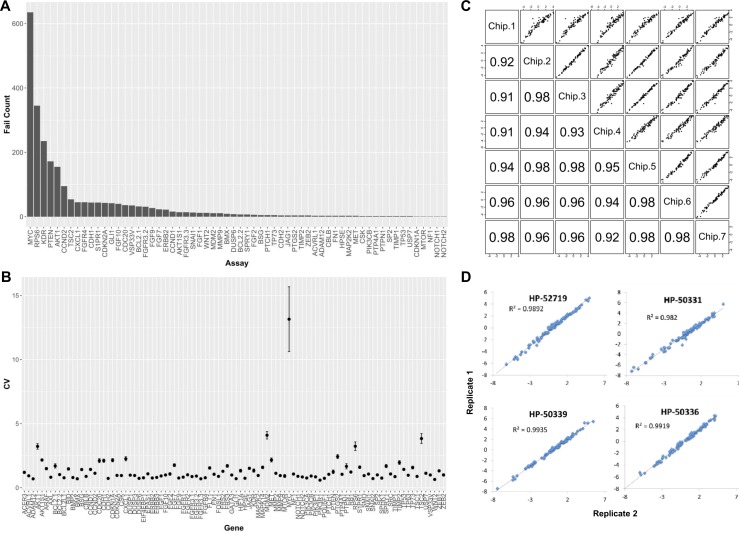
Technical validation of the bladder cancer panel. (A) Bar chart reflecting the failure counts for each of the assays on the bladder cancer panel, as determined by the number of times an assay failed to produce a measurable signal above background in 664 attempted measurements. (B) CV calculations from triplicate experimental measurements using standard deviation over the mean expression values for each of the assays on the panel. (C) Chip-to-chip data reproducibility for high quality control uRNA samples from different runs. (D) Run to run data reproducibility for archival tissues, as seen for two representative FFPE-derived RNA samples run on different days.

To assess the robustness of our panel and its reproducibility in measuring gene expression we conducted a series of quality control experiments. First, we carried out serial dilutions on high quality universal RNA (uRNA) to evaluate the performance of each of the assays, redesigning primers when necessary, to achieve linear standard dilution curves for all tests (S4 Fig). During this process, the FGF23, ERBB3, USP7, BMP2, DUSP3, SRC, CSK, MTOR, GLI1, and MET assays did not yield satisfactory standard dilution curves the first time and were re-designed for optimal performance. To assess performance of the QRT-PCR assays on our panel, we ran in triplicate high quality uRNA control samples and lower quality RNA derived from 204 FFPE clinical tissues and counted the number of times an assay failed to produce a measurable signal above background (Fig 2A). Five assays (RPS6, KDR, PTEN, AKT1, CCND2) had at least 100 failures over 664 attempted measurements (>15% failure rate) (Fig 2A). However, even for RPS6, which was the poorest performing of these five assays, we observed measurable signal above background in 319/664 attempts (48% success rate). Notably, the MYC assay had 635/664 failures (96% failure rate) and was the poorest performing assay on the panel (Fig 2A).

Next, we evaluated assay variability by calculating coefficient of variation (CV) over the three replicate measurements that were obtained from our quality control experiments for each assay and sample combination. CV was calculated as the ratio of standard deviation over the mean expression measurement. The average CV for all assays on the panel was under 5% except for the MYC assay, which had an average CV of 13% when analyzing cell line- and FFPE tissue-derived RNA (Fig 2B). Notably, a CV value of 22% was observed for the MYC test when analyzing high quality uRNA samples (S5 Fig), suggesting that the poor performance was an assay- rather than a RNA quality-related issue. Because of the high failure rate and high CV values obtained with the MYC assay, this test was dropped from all subsequent analyses.

We also assessed data reproducibly when running high quality uRNA and lower quality RNA from FFPE tissues on different runs from different days. We noted a high degree of chip-to-chip data reproducibility for each gene on our panel when we ran uRNA control samples as part of seven independent runs (Fig 2C, R^2^ range = 0.91–0.98). Furthermore, we ran replicate FFPE-derived RNA samples on different days and observed a high degree of data concordance for each of the genes on the panel (Fig 2D, R^2^ range = 0.98–0.99). The results from our qualitative and quantitative assessments suggest that all assays on the panel, with the exception of the MYC test, were robust and yielded reproducible data when analyzing both high quality uRNA and lower quality RNA from FFPE tissues.

### Application of the bladder cancer panel in the basal/luminal characterization of FFPE tissues from a novel clinical cohort

To begin to assess the utility of our panel in the basal/luminal characterization of FFPE tissues, we analyzed a set of 204 samples comprised of NMIBCs, MIBCs, as well as lymph node and distal METs (Table 2, S3 Table). We first examined how our samples compared to those from several public datasets with respect to basal/luminal transcriptional features. As expected and described earlier in Fig 1B, hierarchical clustering of samples from the four public datasets [10, 12–14] using median centered expression from probes corresponding to genes on our panel revealed a clear separation between basal and luminal samples (S6A Fig). Notably, luminal samples from the Kim dataset clustered with the basal samples from the three other datasets and were outliers in this analysis (S6A Fig). MIBCs from our cohort clustered alongside basal samples from all four public datasets (S6A Fig), consistent with a basal-like transcriptional profile. In our dataset, most NMIBCs clustered with luminal samples and, thus, were likely to be luminal-like in nature (S6A Fig). To our knowledge, there have been no previous reports on the basal/luminal features of METs. METs from our cohort clustered with basal samples from the public datasets (S6A Fig).

**Table 2:** Clinicopathologic features of a novel bladder cancer cohort.

	Number	Percentage
**Age (at diagnosis)**		
Median	65	
Range	35–91	
Standard Deviation	11.51	
**Gender**		
Male	164	79%
Female	43	21%
**Tissue Specimens**		
Primary Bladder Cancer (T0-T4)	154	75.5%
Non-Muscle-Invasive(T0, T1, Tis)	93	45.5%
Muscle-Invasive (T2, T3)	61	30.0%
Metastatic Bladder Cancer (T4)	53	26.0%
Lymph Nodes	45	22.0%

**Fig 3:**
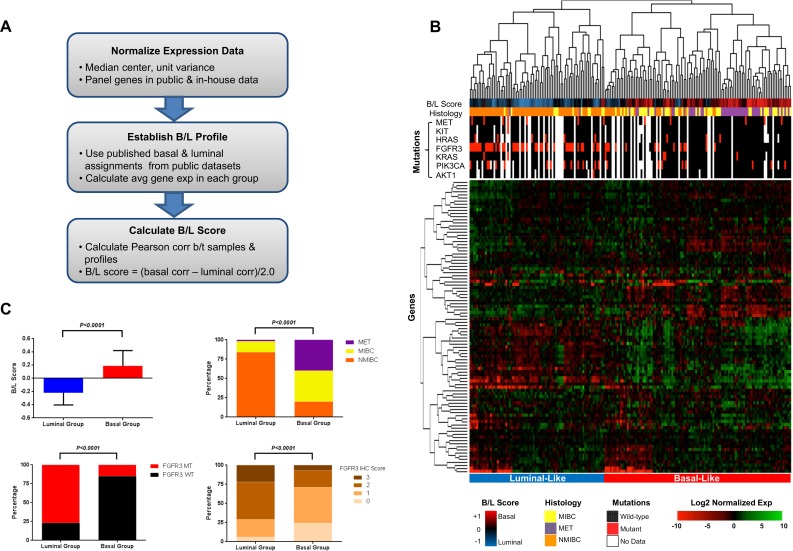
Molecular characterization of a novel cohort of FFPE tissues using the bladder cancer panel. (A) Methodology used for computing basal/luminal signatures from public data and then applying these signatures to bladder panel data to determine basal/luminal similarity of samples from a novel clinical cohort. (B) Hierarchical clustering (average-linkage, 1 –Pearson correlation distance metric) of 204 FFPE samples based on bladder cancer panel gene expression and corresponding B/L scores, histology, and mutational status of cancer-relevant genes. (C) Statistical analysis of the B/L scores (top left panel), distribution of NMIBCs, MIBCs, and METs (top right panel), prevalence of *FGFR3* mutations (bottom left), and FGFR3 IHC scores (bottom right) in samples from the transcriptionally-defined luminal- and basal-like clusters in Fig 3B.

We next established a method for calculating basal/luminal similarity scores using transcriptional signatures from our FFPE samples. We began by median-centering the expression of all probes corresponding to the genes on our panel in public datasets and calculated an average gene expression value in basal and luminal groups, as assigned by Choi and Damrauer and colleagues [10, 14] (Figs 3A and S6B). This allowed us to obtain an average view of the expression of each of these genes in the luminal and basal groups from the public datasets (S6B Fig). For example, *FGFR3* had the highest average expression value in luminal samples than any other gene on our panel, and the average *FGFR3* expression level was one of the lowest in basal samples from the public datasets (S6B Fig). We then derived basal/luminal (B/L) similarity scores for each of the 204 FFPE samples by calculating their correlation to the public basal and luminal profiles (Fig 3A). Hierarchical clustering of our FFPE samples revealed two main branches with distinct patterns of gene expression: 1) a right branch under which the majority of luminal-like samples with low B/L similarity scores were co-clustered; and 2) a left branch that was comprised mostly of basal-like samples with high B/L scores (Fig 3B). Statistical analysis confirmed a significantly lower B/L score for samples under the left branch of the clustergram, which is consistent with luminal-like status, and a significantly higher B/L score consistent with basal-like status for specimens under the right arm of the hierarchical tree (Fig 3B and 3C, top left panel; *P<0*.*001*). We observed a significantly higher fraction of NMIBCs in the luminal-like compared to the basal-like group (Fig 3B and 3C, top right panel; *P = 0*.*0001*), consistent with the notion that NMIBCs from our cohort are mostly luminal-like in nature. MIBCs were represented in both basal- and luminal-like groups; however, a significantly higher proportion of MIBCs from our series was observed in the basal-like group (Fig 3B and 3C, top right panel; *P = 0*.*0001*). Interestingly, almost all METs clustered under the basal-like arm of the hierarchical tree, suggesting that the majority of the METs in our cohort exhibited a basal transcriptional profile (Fig 3B and 3C, top right panel; *P = 0*.*0001*).

One of the characteristic features of NMIBCs is that they carry somatic activating mutations in *FGFR3* in ~60–80% of the cases, and a much lower frequency of ~15% has been reported in MIBCs and METs [19, 25, 27, 33–37]. We assessed the mutational status of *FGFR3* and other cancer-relevant genes in samples from cohort using a custom allele-specific PCR panel [19, 33, 34, 36–38]. We found that >75% of the tumors that co-clustered under the luminal-like label carried *FGFR3* mutations, a significantly higher fraction than the <20% *FGFR3* mutation rate we observed in samples from the basal-like group (Fig 3C, bottom left panel; *P<0*.*0001*). Mutations in *FGFR3* have been reported to drive higher expression levels of the RTK [19]. Consistent with this finding, we noted significantly higher levels of FGFR3 protein, as measured by IHC, in samples from the luminal-like compared to the basal-like groups (Fig 3C, bottom right panel; *P<0*.*0001*). We did not observe significant associations between basal- or luminal-like status and any of the other mutations on our panel (Fig 3B). These findings suggest that our bladder cancer panel can classify FFPE samples into luminal- and basal-like groups. The enrichment of *FGFR3* mutations and up-regulation of the RTK on the protein level that we observed in the luminal-like samples and the absence of these features in basal-like samples provides molecular confirmation of the basal/luminal nature of the samples in our cohort.

## Discussion

Transcriptional analyses have provided valuable insights into the molecular underpinnings of bladder cancer [11–15, 31, 39, 40]. However, the majority of these studies relied on the use of frozen tissues that yield high quality nucleic acids and are not well-suited for the characterization of poor quality RNA from FFPE samples. Our microfluidics-based bladder cancer panel is custom designed for robust transcriptional characterization of FFPE tissue-derived RNA.

An intriguing finding in the bladder cancer field was that MIBCs were reminiscent of the basal and luminal classes of breast cancers, and that they defined different disease subtypes that were associated with distinct clinical outcomes [10, 14]. The genes on our panel were able to accurately segregate samples from four public datasets into basal and luminal groups [10, 12–14], pointing to the possibility of using our platform for detection of basal/luminal status in FFPE tissues from the clinic. The content of our panel performed as well as the BASE47 classifier [10] in stratifying bladder cancers into basal and luminal subtypes. Cross-validated misclassification rates were comparable between our panel and the BASE47 classifier for all four public datasets tested and ranged from 0–16%.

There was minimal overlap between the genes on our panel and those from the public basal/luminal signatures [10, 14]. This suggests that the basal/luminal features of bladder cancers include broad-ranging differences in gene expression beyond those described by Damrauer and Choi *et*. *al*. [10, 14]. The content of our panel provides an additional advantage in that it measures the expression of genes belonging to bladder-cancer relevant pathways that the public signatures might not include. As such, it may provide a preliminary assessment of these pathways in archival clinical samples. Our panel includes genes that are relevant in indications beyond bladder cancer. Future studies may be warranted to assess the ability of these genes to distinguish between basal/luminal tumors from other indications. Although our bladder panel performed well when classifying bladder cancers into basal/luminal subtypes, it was not as robust in identifying P53-like tumors. Therefore, we recommend using our panel for the basal/luminal but not p53-like characterization of bladder cancers. Given the unique intrinsic features of p53-like tumors, the stability of the p53-like subtype should be examined in additional cohorts in future studies.

On a technical level, 85/90 assays on our panel performed robustly when analyzing high quality uRNA and lower quality RNA derived from FFPE tissue, as evidenced by failure rates of <15% and a CV of <5% across all samples. Five assays (RPS6, KDR, PTEN, AKT1, CCND2) had failure rates of >15%; however, even the worst performing assay (RPS6) had a success rate of 48%, indicating that these assays were still informative in about half of the samples. We excluded the MYC assay from further analysis because it was the worst performing test on the panel with a 96% failure rate, and it also exhibited a CV of 22% when we analyzed high quality uRNA samples and a CV of 13% when using RNA from FFPE tissues. When we assessed data reproducibly we observed a high degree of data concordance for the assays on our panel for uRNA replicates from independent runs. Furthermore, a high degree of data concordance was observed for each of the assays when we ran replicate FFPE-derived RNA samples on different days. Our qualitative and quantitative assessments suggest that our panel can provide robust and reproducible results when analyzing archival clinical samples.

We utilized our panel in the characterization of a novel cohort of 204 FFPE tissues and were able to identify samples that were transcriptionally similar to the recently described basal and luminal bladder cancer subtypes [10, 14]. By deriving a basal/luminal scoring system using the genes on our panel, we confirmed previous findings using our FFPE cohort that MIBCs can exhibit basal or luminal transcriptional patterns [10, 14]. To our knowledge, this is the first report on the basal/luminal features of METs. We found the majority of METs from our cohort to have a basal-like transcriptional profile. There has been a recent controversy around the luminal/basal make-up of NMIBCs [16, 17]. McConkey and colleagues found the majority of their chemotherapy-naïve NMIBCs to be luminal [17]. On the other hand, in a recent study Hedegaard *et*. *al*. showed that NMIBCs from their cohort were mostly basal in nature [16]. The basal-/luminal-like features may vary depending on the spectrum of tumors included in different cohorts. In our sample series we found the majority of NMIBCs to be luminal-like. Mutational status of the *FGFR3* gene and expression levels of the RTK by IHC provided molecular confirmation of the luminal and basal status of our samples, by virtue of the high frequency of *FGFR3* mutations and high expression levels of the protein that was detected in luminal samples and the low frequency of mutations and protein expression levels that was observed in the basal samples [10, 14, 19]. Differences in the etiologies of NMIBCs might be one reason for the observed differences in the basal/luminal make up of samples from different cohorts. Additional studies into to the basal and luminal characteristics of NMIBCs will be required to resolve these discrepancies.

This study provides proof-of-concept that our microfluidics-based bladder cancer panel can robustly classify FFPE tissues into the clinically-relevant basal and luminal disease subtypes. Further validation efforts on additional archival tissues will be required before this panel can be implemented in the clinic. By making the panel publically available at this point in time we provide the field with the opportunity to apply this rapid, robust, and cost effective approach in the basal/luminal characterization of FFPE clinical samples. This approach offers additional important advantages over global transcriptional profiling for basal/luminal classification, including relatively simple data analysis and suitability for analyzing FFPE-derived RNA, which is often the only available substrate when working with clinical specimens.

## Material and Methods

### Selection of bladder cancer panel genes

The bladder cancer panel is built on a commercially available microfluidics platform, and this work is not intended for marketing or commercialization of any product. Selection of gene content was based on a comprehensive review of the literature and prioritization of pathways and related genes that have been shown to be involved in bladder cancer development [12, 18, 20, 23, 26, 30] (S1 Table, S1 Fig). Due to the limitation in the number of assays that could be accommodated on the panel and the need to include controls for data QC and normalization (2 housekeeping genes), only 91 unique bladder cancer-relevant genes could be selected. Due to poor technical performance, the MYC assay was dropped from all analyses, bringing the total number of genes on the panel down to 90. Panel genes were binned into three main categories (Development and EMT, FGFR, RTK, MAPK, and PI3K, and TP53, genome stability, and cell cycle genes) based on Ingenuity® pathway analysis (Fig 1A).

### Analysis of public datasets

Analyses were performed using the R statistical language (R version 3.2.2) with the Bioconductor library [41] (version 3.1) to support microarray analysis, unless noted otherwise. Several public datasets were used to assess the ability of the 90 genes on our panel to capture basal/luminal status. Public data sets were downloaded from the Gene Expression Omnibus (GEO) (http://www.ncbi.nlm.nih.gov/geo/) using the GEOquery R library (version 2.34.0). Expression data was normalized using median polish [42] (medpolish command from the R stats library). The ability of the 90 genes on our bladder panel to identify basal-like and luminal-like samples based on transcriptional profiling data was assessed in four literature data sets (GSE32894, GSE5287, GSE13507, GSE48075). Basal or luminal classification was as described by Damrauer *et*. *al*. [10].

### Cross-validation classifier analysis of public data sets

The genes on our panel were evaluated for their ability to distinguish between basal and luminal subtypes in public data sets. Using the CMA R library (version 1.28.1) we performed 5-fold cross-validation, training a diagonal linear discriminant analysis (DLDA) classifier [32] using all genes and 80% of randomly selected samples. The classifier performance was then evaluated on the remaining 20% of the samples for validation. This procedure was repeated 5 times until all samples had been included in the test set one time. Misclassification rates were evaluated for each fold and mean rates and standard errors were calculated. To identify the highest performing subsets of genes on our panel in distinguishing between basal and luminal subtypes, we repeated the cross-validation process but incorporated a gene sub-selection process at each iteration. Specifically, with each iteration the top n genes were selected from the panel based on their differential expression in the 80% training samples from each public dataset. Differential expression was evaluated using a Welch’s t-test. The number of selected genes was n = 10, 20, 30, 40, 50, 60, 70 and 80, and mean misclassification rates and standard errors were calculated in each case.

### Calculating basal/luminal expression profiles from public datasets

The Damrauer discovery dataset [10] (N = 30, Affymetrix Human Genome U133A Array) was downloaded from the NCBI Gene Expression Omnibus (GSE5287). Affymetrix probe sets corresponding to genes on the bladder panel were used. The remaining subset of log-transformed expression data were then mean centered and normalized to unit variance. Average gene expression values were calculated for the 90 genes across the 12 samples classified as luminal-like and 18 samples classified as basal-like (classifications received through author correspondence) to produce basal and luminal expression profiles. This process was repeated for three other public datasets (GSE32894, GSE13507, GSE48075).

### Tissues and histopathology

A collection of 204 FFPE bladder cancer samples was obtained from Cureline, Inc. (South San Francisco, CA) following approval of the Ethics Committee of Saint Petersburg City Clinical Oncology Hospital and appropriate confirmation of written informed consent, or from The MT Group (Van Nuys, CA) following IRB approval (http://www.sterlingirb.com). The clinical samples were of a ~80%/20% male/female ratio, and the proportions of NMIBCs (T0, T1), MIBCs (T2, T3) and metastases were 45%, 30%, and 26%, respectively (Table 2, S3 Table). Hematoxylin & Eosin-stained (H&E) sections were evaluated by two independent pathologists (DF and OF). Primary tumors were divided into NMIBCs (T0, T1, Tis) and MIBCs (T2 and T3), and bladder cancer metastases were either from lymph nodes or distal organs (Table 2, S3 Table). Tumor areas for cases with less than 70% neoplastic cellularity were marked by a pathologist for macro-dissection and subsequent tissue lysis for nucleic acid preparation. Overall, tumor content ranged from 70–90%. RNA and DNA were extracted from macro-dissected samples as previously described [38, 43].

### Expression analysis of FFPE tissues

Gene expression analysis was carried out on RNA extracted from macro-dissected FFPE tissues using the High Pure FFPE RNA Micro Kit (Roche Diagnostics, Indianapolis, IN) after de-paraffinization with Envirene, as described previously [44]. RNA was quantified using NanoDrop® (Thermo Scientific, Willmington, DE), and the average 260/280 reading was 1.74. Due to the limiting amounts of available RNA it was not possible to assess the quality of the RNA electrophoretically to calculate RNA integrity number (RIN) values. Gene expression analysis was performed on patient specimens starting with 100ng total RNA that was reverse-transcribed to cDNA and pre-amplified in a single reaction using Superscript III/Platinum Taq and pre-amplification reaction mix (Invitrogen, Carlsbad, CA). All Taqman primer/probe sets were included in the pre-amplification reaction at a final dilution of 0.05x original Taqman assay concentration (Applied Biosystems, Foster City, CA). The thermocycling conditions were as follows: 1 cycle of 50°C for 15 min, 1 cycle of 70°C for 2 min, then 14 cycles of 95°C for 15 sec and 60°C for 4 min. Pre-amplified cDNA was diluted 2-fold and then amplified using Taqman Universal PCR MasterMix (Applied Biosystems, Foster City, CA) on the BioMark BMK-M-96.96 platform (Fluidigm, South San Francisco, CA) according to the manufacturer’s instructions. All samples were assayed in triplicate. Cycle threshold (Ct) values were converted to relative expression using the ΔCt method [44], where ΔCt was the mean of the target gene minus the geometric mean of reference genes calculated for the respective patient specimen. For genes assessed on the bladder cancer panel, Cycle threshold (Ct) values were normalized using median polish [42] (medpolish command from the R stats library). Hierarchical clustering was carried out on normalized data with the average-linkage method using 1 –Pearson correlation as a distance metric using the heatmap.2 function from the gplots R library (version 2.17.0) and subsequently visualized using R [45].

### Mutation analysis

Mutation analyses were carried out on genomic DNA extracted from macro-dissected FFPE tissues using the QIAamp FFPE kit (Qiagen, Valencia, CA) after de-paraffinization with Envirene [15]. Mutations in *PIK3CA*, *EGFR*, *KRAS*, *NRAS*, *HRAS*, *FGFR3*, *MET*, *BRAF*, *KIT*, *AKT1*, *FLT3* were detected using mutation specific qPCR as described previously [38].

### Immunohistochemistry

IHC was performed on 4-μm thick FFPE tissue section using the Ventana Discovery XT Autostainer platform (Ventana Medical Systems Inc, Tucson, AZ). For the detection of FGFR3, the slides underwent pretreatment using CC1 extended antigen retrieval followed by anti-FGFR3, clone 15C3 (Genentech), primary antibody diluted to 1 μg/mL and incubated for 60 minutes at room temperature. For amplification of FGFR3 signal, an unconjugated rabbit-anti-mouse linker antibody (Jackson Immunoresearch, West Grove, PA) was applied at 1 μg/mL and incubated for 32 minutes at room temperature. This was followed by an anti-rabbit-OMNIMAP-HRP kit (Ventana Medical Systems Inc, Tucson, AZ. Catalog no. 760–4310). Sections were counter stained with hematoxylin, dehydrated, cleared and cover-slipped for viewing.

### Calculation of basal/luminal similarity scores and statistical analysis

Basal/luminal similarity scores for FFPE samples were determined by first deriving basal/luminal expression profiles from the public Damrauer discovery data set, as previously described [10]. Next, basal and luminal Pearson correlations between each profile and each mean-centered, unit variance normalized FFPE sample were determined. These two correlation scores were combined to produce an overall B/L similarity score: B/L score = (basal profile correlation–luminal profile correlation)/2.0. The B/L score had a range of +1.0 to -1.0, with scores above zero indicating a basal-like sample, and scores below zero indicating a luminal-like sample. For the significance of differential mutation frequency between clusters, Fisher's exact T-tests were performed. Gene differential expression was calculated using the multi t-test (mt.maxT) from the multtest library (version 2.26) in Bioconductor [41] (S2 Table). The test statistic columns show the two-sample Welch t-statistic (unequal variances). This statistic was calculated as the fold change difference divided by the sum of the basal and luminal variances weighted by number of samples in each group. The sign of the statistic reflected the fold change direction, where a positive score implied the gene is more highly expressed in basal samples. Values were left blank in the table when a gene from the 90-gene panel was not present in the literature data set.

## Supporting Information

S1 Fig Schematic diagram of the selection criteria for bladder cancer panel genes. (TIF)

S2 Fig Cross-validation misclassification rates as a function of the number of genes used in the classifier. Number of classifier genes ranging form 10–80 was used to train a DLDA classifier on 80% of samples from each public data set, and misclassification rates were calculated on the remaining 20% of the samples. Procedure was repeated 5 times through cross-validation to calculate average misclassification rates and to estimate standard errors.
(TIF)

S3 Fig Hierarchical clustering of samples from the Choi cohort based on the signals of probes corresponding to the genes on the bladder cancer panel. Mutation data based on the Choi study is also provided.
(TIF)

S4 Fig Linear performance of six representative assays and their raw CT values with increasing input amounts of universal RNA (uRNA) controls. (TIF)

S5 Fig CV calculations using triplicate experimental measurements from analysis of high quality uRNA samples. Standard deviations over the mean expression values was calculated for each of the assays on the panel.
(TIF)

S6 Fig Calculation of basal/luminal similarity scores based on the content of the bladder cancer panel. (A) Heatmap showing unsupervised clustering of genes and samples from public data sets, as well as NMIBCs, MIBCs, and METs from a novel FFPE tissue cohort. White blocks represent genes that are not found in the respective data sets. (B) Basal profile (red bars) and luminal profile (blue bars) for bladder cancer panel genes calculated from the Damrauer discovery samples. Mean-centering and unit variance normalization was applied to the log-transformed expression values, and mean log normalized expression levels were calculated independently for basal and luminal sample groups to form the final profiles.
(TIF)

S1 Table Genes comprising the custom bladder cancer microfluidics panel and corresponding assays. (XLSX)

S2 Table Bladder cancer panel genes significanly differentially expressed between basal and luminal samples from the Choi discovery cohort. (XLSX)

S3 Table Bladder cancer cohort clinical & histological attributes and FGFR3 mutations and IHC data. (XLSX)
