# Supplementary figures and images for: Development and Application of a Microfluidics-Based Panel in the Basal/Luminal Transcriptional Characterization of Archival Bladder Cancers
Source: PLoS One. 2016 Nov 15;11(11):e0165856. doi: 10.1371/journal.pone.0165856 (PMC5112874; doi:10.1371/journal.pone.0165856)

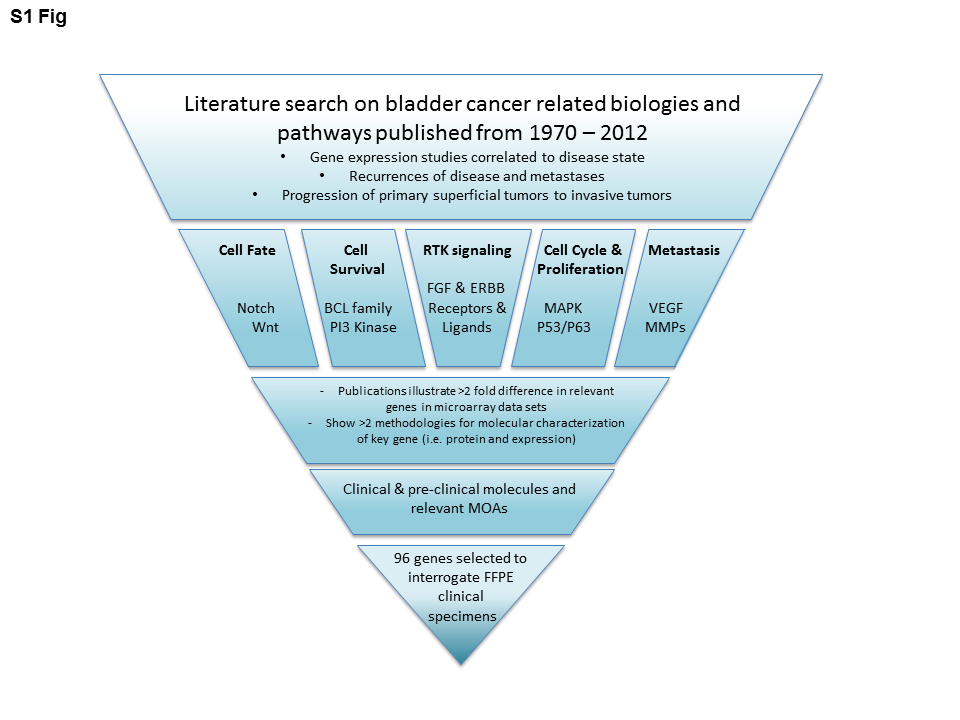

Supplement: S1 Fig — (TIF) [file pone.0165856.s001.TIF]

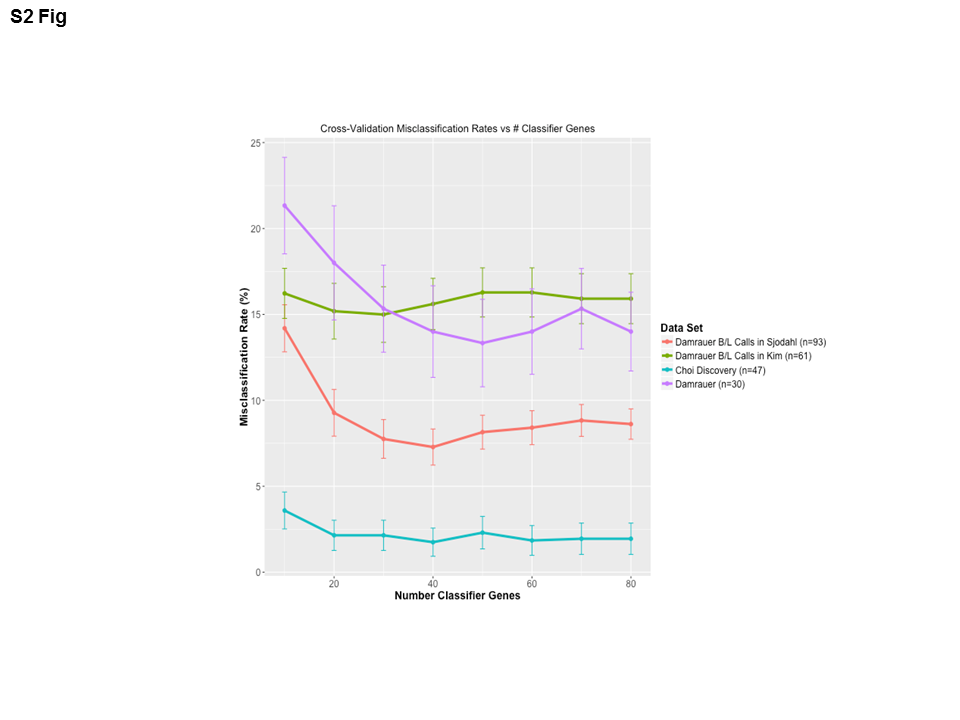

Supplement: S2 Fig — Number of classifier genes ranging form 10–80 was used to train a DLDA classifier on 80% of samples from each public data set, and misclassification rates were calculated on the remaining 20% of the samples. Procedure was repeated 5 times through cross-validation to calculate average misclassification rates and to estimate standard errors. (TIF) [file pone.0165856.s002.TIF]

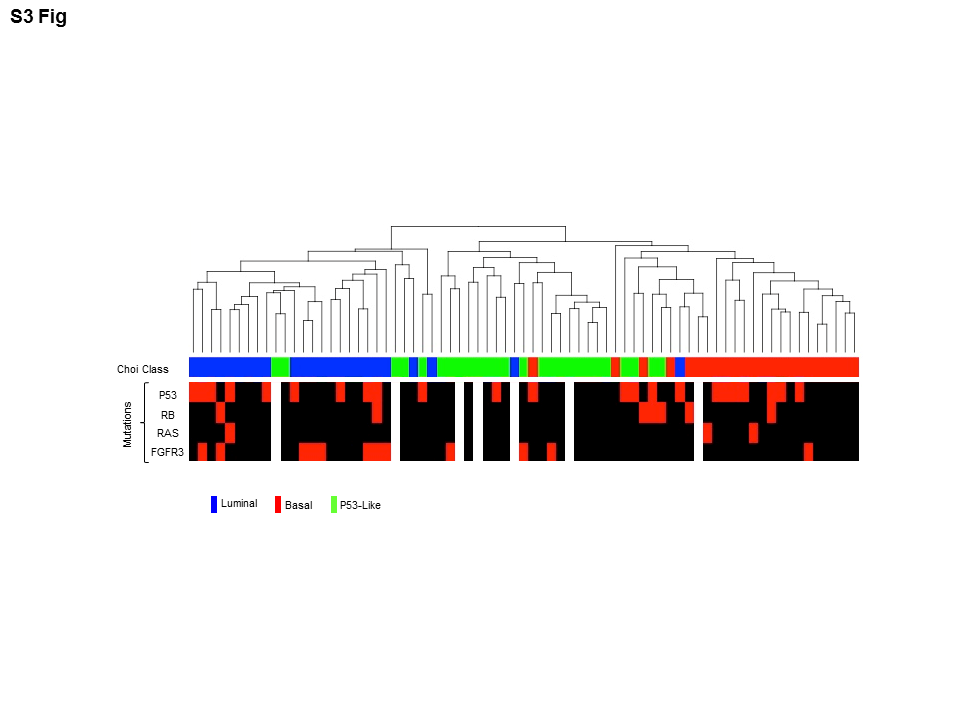

Supplement: S3 Fig — Mutation data based on the Choi study is also provided. (TIF) [file pone.0165856.s003.TIF]

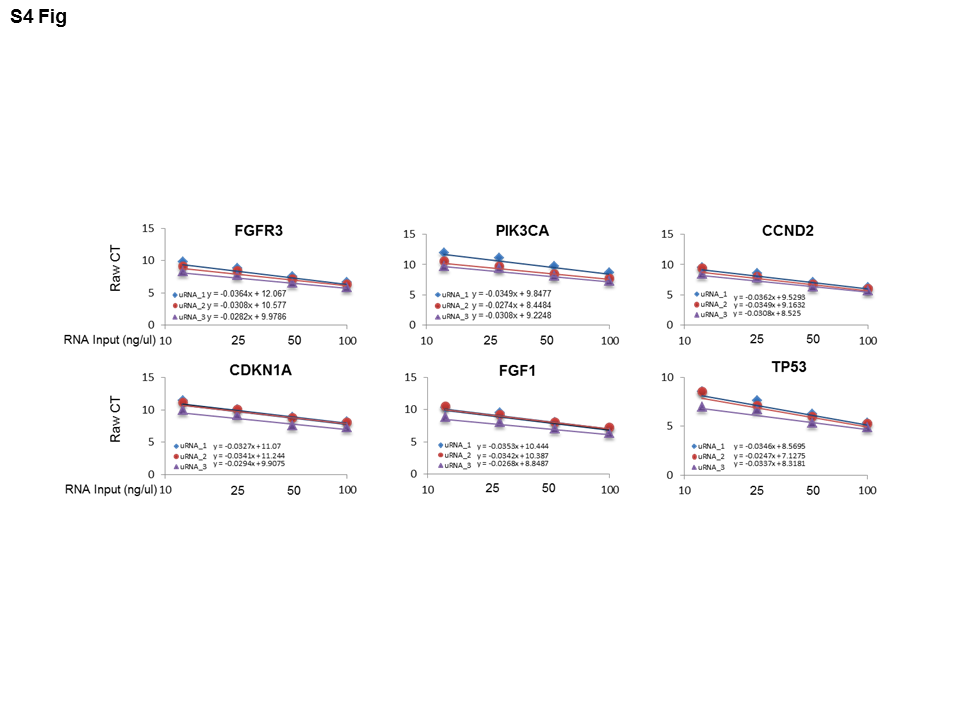

Supplement: S4 Fig — (TIF) [file pone.0165856.s004.TIF]

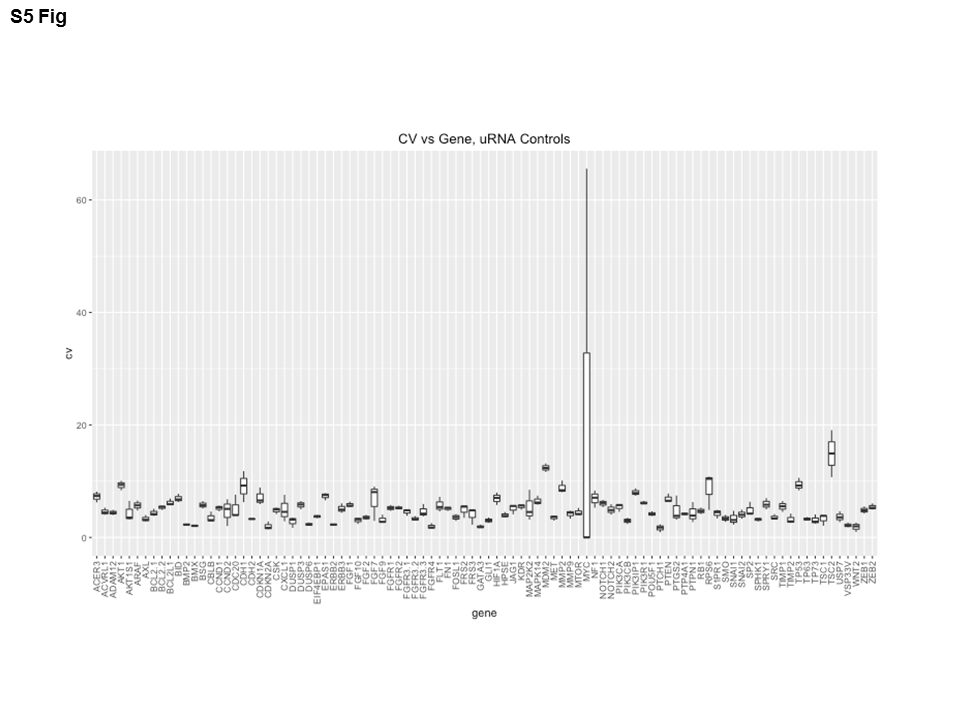

Supplement: S5 Fig — Standard deviations over the mean expression values was calculated for each of the assays on the panel. (TIF) [file pone.0165856.s005.TIF]

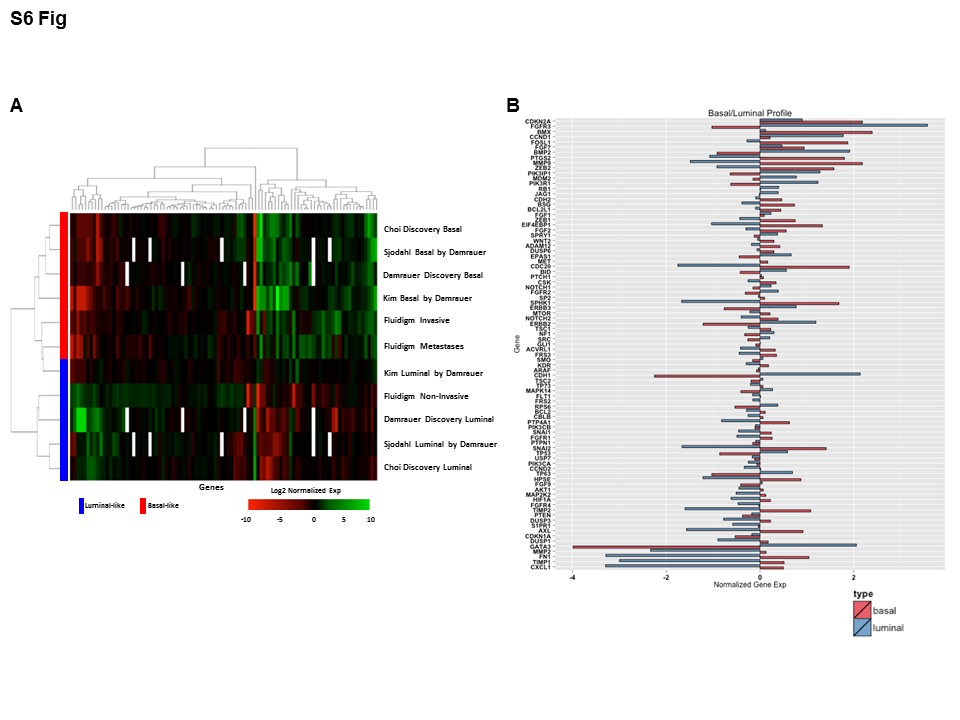

Supplement: S6 Fig — (A) Heatmap showing unsupervised clustering of genes and samples from public data sets, as well as NMIBCs, MIBCs, and METs from a novel FFPE tissue cohort. White blocks represent genes that are not found in the respective data sets. (B) Basal profile (red bars) and luminal profile (blue bars) for bladder cancer panel genes calculated from the Damrauer discovery samples. Mean-centering and unit variance normalization was applied to the log-transformed expression values, and mean log normalized expression levels were calculated independently for basal and luminal sample groups to form the final profiles. (TIF) [file pone.0165856.s006.TIF]
